# Microhardness of different resin cement shades inside the root canal

**DOI:** 10.4317/medoral.17802

**Published:** 2012-02-09

**Authors:** Valeria Vignolo, Maria V. Fuentes, Miguel A. Garrido, Jesús Rodríguez, Laura Ceballos

**Affiliations:** 1 DDS, Department of Stomatology. Rey Juan Carlos University; 2 DDS, PhD, Assistant Professor, Department of Stomatology. Rey Juan Carlos University; 3 PhD, Associate Professor, Department of Material Sciences and Engineering. Rey Juan Carlos University; 4 PhD, Full Professor, Department of Material Sciences and Engineering. Rey Juan Carlos University; 5 DDS, PhD, Associate Professor, Department of Stomatology. Rey Juan Carlos University

## Abstract

Objectives: To compare microhardness along the root canal post space of two resin cements in different shades and a dual-cure resin core material. Study Design: Root canals of 21 bovine incisors were prepared for post space. Translucent posts (X•Post, Dentsply DeTrey) were luted using one the following resin luting agent: Calibra (Dentsply DeTrey) in Translucent, Medium and Opaque shades, RelyX Unicem (3M ESPE) in Translucent, A2 and A3 shades and the dual-cure resin core material Core•X flow. All materials were applied according to manufacturers’ instructions and were all photopolymerized (Bluephase LED unit, Ivoclar Vivadent, 40s). After 24 hours, roots were transversally cut into 9 slices 1 mm thick from the coronal to apical extremes, three corresponding to each root third. Then, VHNs were recorded (100 gf, 30 s) on the resin luting materials along the adhesive interface in all sections. Data were analyzed by two-way ANOVA and SNK tests (α=0.05). Results: A significant influence on microhardness of resin luting material in their respective shades (p<0.001), root third (p<0.001) and interactions between them was detected (p<0.001). RelyX Unicem cement showed the highest microhardness values and Calibra the lowest, regardless of the shade selected. All resin luting materials tested exhibited a significantly higher microhardness in the cervical third. Conclusions: Microhardness of resin luting agents tested inside the canal is dependent on material brand and resin cement shade seems to be a less relevant factor. Microhardness decreased along the root canal, regardless of the shade selected.

** Key words:**Cement shade, degree of conversion, dual-cured resin cements, fiber posts, microhardness, root thirds.

## Introduction

>Nowadays, translucent fiber reinforced composite posts constitute the first option to restore non-vital teeth with an excessive loss of coronal structure (1). Besides good esthetics, their main advantage is that their elastic modulus is similar to dentin (2), inducing a favorable distribution of stress which prevents root fractures (1).

In order to achieve this homogeneous biomechanical unit, the use of resin cements is utterly necessary to lute the posts into the roots. Clinicians can choose among resin cements that require the previous application of an adhesive system, self-adhesive resin cements or even dual-cure resin core materials (3).

In all cases, the luting agent selected must be a dual-cure resin material in order to compensate light attenuation, as most of the posts are cemented to an extent of 8 to 10 mm (4,5). However, the polymerization of some dual-cure resin cements has been reported to be mainly dependent on light exposure (6), whilst the chemical-curing method, when acting alone, is considered to be slower, less effective and not capable of compensating the attenuation of light (6,7).

Therefore, the use of translucent fiber posts has been also recommended as they transmit light to deeper depths allowing the activation of light-polymerizing components of the dual-cure resin cements (4,6,8) and, thus, improving their degree of conversion (6).

The polymerization efficacy of resin materials has been also related to characteristics of the material itself, such as chemical composition, filler particle size, and shade and translucence (9,10,11). The darker shades have been reported to exhibit a reduction in light transmission not curing as deeply as lighter shades (12,13). Besides, more translucent materials are considered to allow better light transmission which would result in a higher degree of conversion. Although this circumstance has been described for restorative resin composites (14), no references have been found regarding this possible effect on resin cements polymerization efficacy when used to lute fiber posts.

An adequate degree of conversion of the resin cement is mandatory to ensure that physical and mechanical properties are good enough to withstand masticatory forces immediately after cementation while maintaining an adequate bonding and dentin sealing (15).

The degree of conversion of resin cements can be assessed by indirect methods such as micro hardness testing. This being a widely accepted method as it presents a good correlation with the spectroscopy approach (7,16). Therefore, the aim of this study was to compare the microhardness along the root canal post space of: a resin cement that requires the previous application of an adhesive system; a self-adhesive resin cement; and a dual-cure resin core material. The later is available in only one shade while the formers were tested in 3 different shades. The null hypotheses tested were: the three resin luting materials exhibit similar micro hardness values regardless of the shade; and micro hardness is uniform along the root canal depth.

## Material and Methods 

Twenty one bovine incisors teeth were sectioned at 16 mm from the root apex to standardize the root length. The pulp was then removed with #90-K files (Maillefer, Ballaigues, Switzerland).

A 12 mm-deep post space was prepared in each root with the drills provided by the manufacturer of the posts (X•Post, Size 3, Dentsply DeTrey, Konstanz, Germany), leaving 4 mm in the apical portion. In all cases, the translucent fiber posts X•Post (Size 3: length 20 mm, tip diameter 0.8 mm and head diameter 1.67 mm, Dentsply DeTrey) were used. They were all cut at 16 mm and cleaned with alcohol after try-in.

Before cementation procedures, the external lateral walls of the roots were painted with two layers of black nail varnish to avoid any external light curing. Afterwards, post spaces were rinsed with 2.5% sodium hypochlorite irrigation and dried with absorbent paper points.

The roots were randomly allocated to seven groups according to the resin luting agent used and its shade. The dual-cure resin materials evaluated were: Calibra in Translucent, Medium and Opaque shades (Dentsply DeTrey), which is a resin cement that uses an etch and rinse adhesive system (Prime & Bond NT, Dentsply DeTrey); RelyX Unicem in Translucent, A2 and A3 shades (3M ESPE, Seefeld, Germany), which is a self-adhesive resin cement and Core•X flow (Dentsply DeTrey) which is a composite core material that can be also used as luting material and available only in one shade and uses an etch and rinse adhesive system (XP Bond, Dentsply DeTrey). Chemical composition of the adhesive systems and resin luting materials tested are shown in ( Table 1). They were all used following manufacturers’ instructions.

**Table 1:**
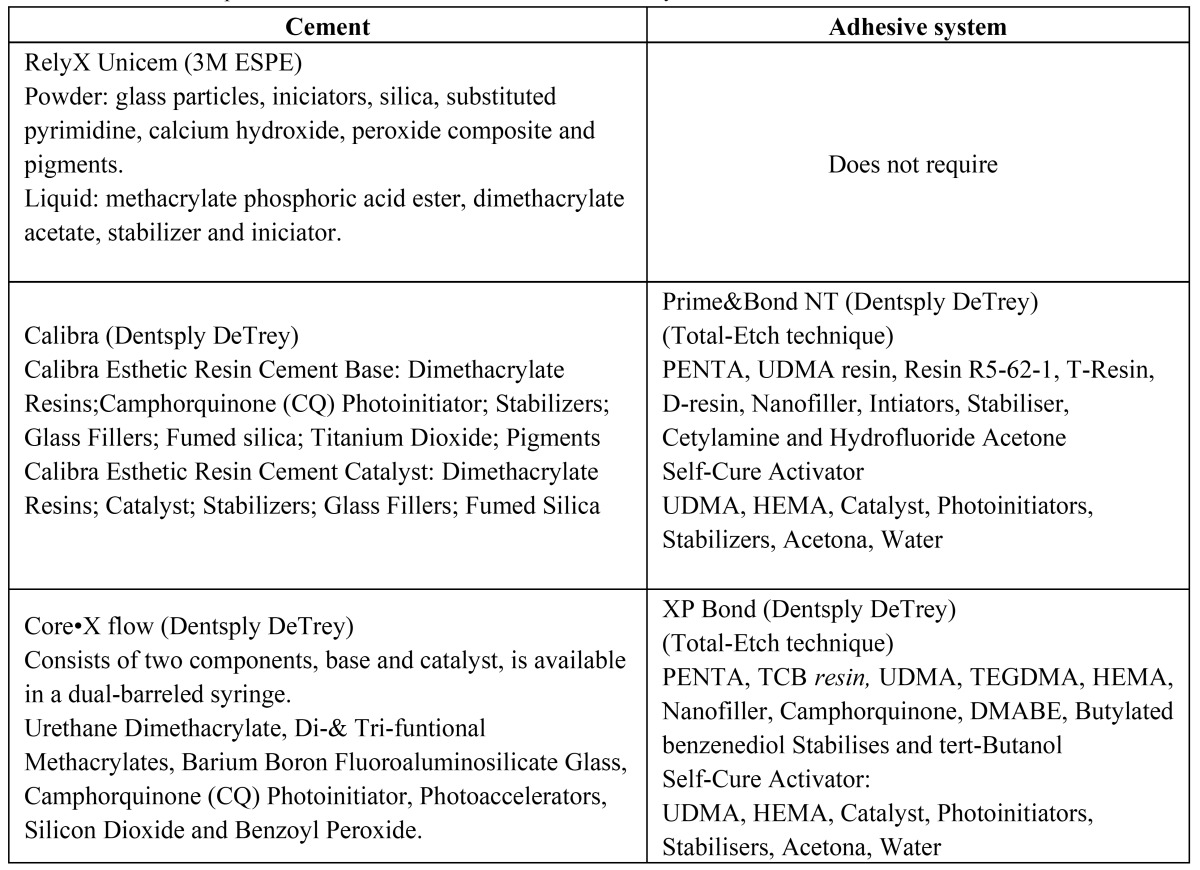
Chemical composition of the resinous cements and adhesive systems.

The resin luting materials were all photopolymerized for 40 s with a LED unit (Bluephase, Ivoclar Vivadent, Schaan, Liechtenstein) applying the high intensity program (1200mW/cm2). The tip of the light unit was kept directly in contact with the coronal end of the post.

Afterwards, specimens were included in resin blocks (Epoxicure Resin-Hardener, Buehler, Lake Bluff, IL, USA) and stored for 24 h in light-proof containers, at 37°C until testing. Roots were then sectioned perpendicular to their long axis with a diamond blade (Accutom-5, Struers, Copenhagen, Denmark), under irrigation. Nine serial slices 1 mm thick were obtained from the coronal to apical extremes, three corresponding to each root third. Surfaces to be analyzed were po-lished with 4000-grit SiC papers for 30 s at 300 rpm (Labopol-5, Struers).

Indentations were performed on the resin luting materials along the adhesive interface between root dentin and the fiber post in all the sections obtained by means of a Vickers digital microdurometer (Buehler 2101, Buehler), applying 100 gf during 30 s. A minimum distance corresponding to the length of two indentations was maintained between indentations and between indentations and the post or the dentin. Hardness was expressed as Vickers hardness number (VHN).

The influence of resin luting agent used considering its shade and the root third was analyzed by two-way ANOVA. Posterior comparisons were performed by Student-Newman-Keuls test. All statistical testing was performed at a pre-set alpha of 0.05 by means of IBM SPSS 18 (IBM Company, Chicago, IL, USA).

## Results

**Figure 1:**
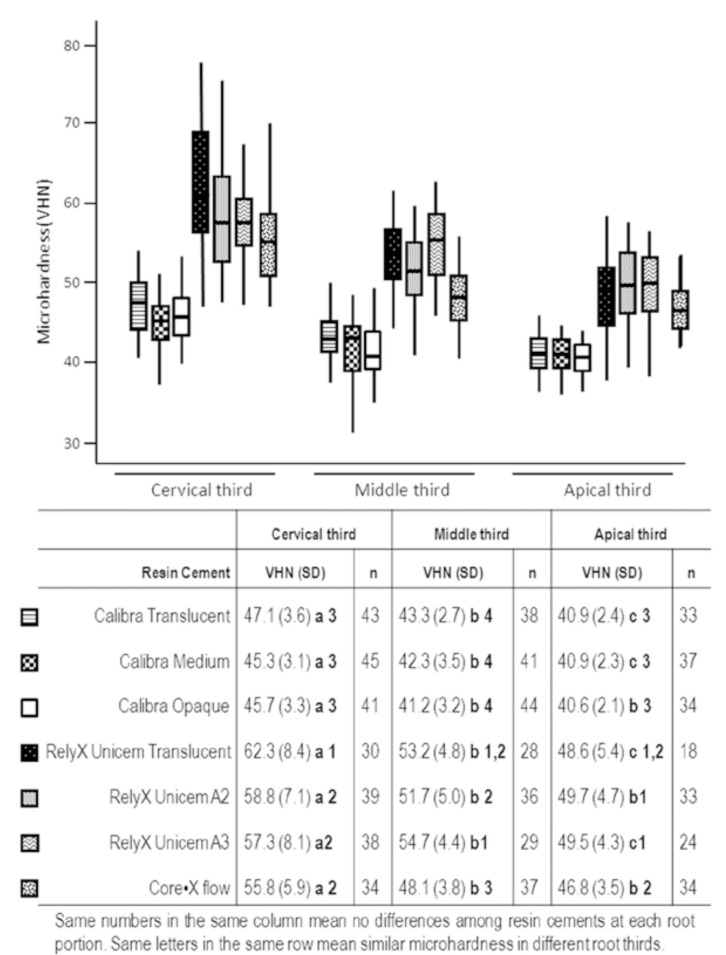
Means VHN and standard deviations determined for the resin luted materials tested along the root canal post space at the cervical, middle and apical thirds.

Fig. 1 shows the mean VHN obtained for each resin luting material and shade evaluated at cervical, middle and apical root third. It must be pointed out that during specimen cutting no displacement of the cemented posts was observed.

According to the results, a significant influence of resin luting material in their respective shades (p<0.001), root third (p<0.001) and interactions between them was detected (p<0.001).

In the three root thirds, the highest microhardness va-lues were attained with RelyX Unicem resin cement and the lowest with Calibra, always and regardless of the shade used. Specimens luted with Calibra showed similar microhardness values for Translucent, Medium and Opaque shades in the three thirds. In contrast, several statistical differences were detected among different shades of RelyX Unicem resin cement. In the cervical third, RelyX Unicem Translucent specimens exhibited micro hardness numbers significantly higher than those obtained with A2 and A3 ones. In the middle third, specimens luted with RelyX Unicem A3 showed statistically higher micro hardness values than those luted with RelyX Unicem A2, but similar in both cases to the ones determined for Translucent shade. In the apical third, no statistical differences were detected among RelyX Unicem shades. Core•X flow micro hardness results were intermediate to the ones attained with the other materials.

Regarding the variation of micro hardness values of each luting material evaluated according to the root third, all of them exhibited a significantly higher micro hardness in the cervical third in comparison with the other two thirds. In the apical third micro hardness values significantly decreased in comparison to the middle third only with RelyX Unicem Translucent and A3 and Calibra Translucent and Medium shades.

## Discussion

The results of the present work indicate that the three resin luting materials tested exhibited significantly different micro hardness values. Thus, micro hardness is dependent on resin luting material brand. RelyX Unicem cement showed the highest micro hardness values and Calibra the lowest, regardless of the shade selected. Moreover, micro hardness of all luting materials evaluated significantly decreased along the root canal. Hence, the null hypotheses formulated must be rejected.

An adequate polymerization of dual-cure post luting cements is desirable to provide mechanical properties good enough to clinically ensure retention of the post (17). Otherwise, all the loads would concentrate on the luting material, the component with the lowest mechanical strength in comparison with dentin and the post (18).

According to literature, micro hardness is a simple testing method that can be correlated to the extent of polymerization (15,19,20). However, care should be taken when comparing different resin materials’ micro hardness as other factors as the nature of the matrix, type of filler, filler load, the quantity of initiators, the amount of inhibitors and the ratio between auto- and light-polyme-rizing components strongly influence the final amount of reacted monomers (7,20).

Self-adhesive resin cements constitute an attractive alternative for post cementation as no dentin pretreatment is required simplifying the procedure and reducing technique and operator sensitivity (20). Specifically, RelyX Unicem has been reported to produce an effective adhesion with dentin despite its very superficial interaction with this tissue, exhibiting similar (21) or even higher bond strength values to root dentin than conventional resin cements (3) and a better sealing ability (22). In the present study, this resin cement attained the highest micro hardness values in comparison with the other two luting materials. Other authors (20,23) have reported a mean micro hardness value somewhat lower for this resin cement (VHN=44). Unlike this study, in both cited works, the luting procedure inside the root canal was not simulated. Although the dominant setting reaction starts with free radical polymerization, phosphoric acidic methacrylates, included in this resin cement, also react with either the basic fillers or the hydroxyapatite of dentin, which may contribute to a higher hardness (24). Regarding the correlation between micro hardness and the degree of cure for this particular cement, information is controversial. While Cadenaro et al. (20) reported a significant correlation between both parameters, in the study by Kumbuloglu et al. (23) the highest microhardness and compressive strength were obtained by this self-adhesive resin cement in comparison with other resin-based luting cements such as Panavia F (Kuraray), Variolink 2 (Ivoclar Vivadent) and RelyX ARC (3M ESPE), but the degree of conversion was, in contrast, the lowest.

The lowest microhardness values were obtained by Calibra resin cement. Previous studies have determined a degree of cure ranging between 59 and 61.5% (25). The lower filler load of this resin cement in comparison with RelyX Unicem may explain the differences in microhardness detected between both materials. Microhardness is related to other mechanical properties as flexural strength (26) and according to our results, in an in vitro study (27), showed that RelyX Unicem obtained higher flexural strength than Calibra after 1-day storage.

In the present study, Core•X flow exhibited intermediate values statistically similar in the cervical third to the ones obtained by RelyX Unicem A2 and A3, and to RelyX Unicem Translucent in the apical third. In the middle third they were intermediate and statistically different to the values determined for Calibra and RelyX Unicem resin cements. It should be mentioned that there is no literature regarding its micro hardness, degree of cure, bonding effectiveness or sealing ability.

According to our results, changes in micro hardness among different shades of the same resin cements have been only significant in cervical and middle root thirds for RelyX Unicem resin cement. In the apical root third, where light intensity inside the canal seems to decrease to levels insufficient for light polymerization, no differences among resin cement shades were detected. Therefore, its influence varies with resin cement brand and root third. In any case, resin cement brand and root third seem to be more relevant than the shade selected for the micro hardness values obtained.

Lighter shades are considered to cure more deeply than darker ones due to an increased capacity for light to penetrate into the bulk of the composite, since pigments in darker shades might absorb more light and reduce its penetration into the resin (12). Nevertheless, translucence seems to be a much more influential predictor of cure depth than any color or shade change in material on curing (9).

In contrast with literature regarding the relevance of shade of resin composites lighter and more translucent shades were not related to significantly higher micro hardness values (13,28). In the present study, only RelyX Unicem Translucent in the cervical third showed higher micro hardness than the same cement in A2 and A3 shades and no differences were detected among Ca-libra shades in any root third.

It must be taken into account that only two resin cements in different shades and only one fiber post brand and one light polymerization procedure were tested. Therefore, other results might be obtained with other resin luting materials. In any case, comparison between different resin luting materials would not be easy as usually shades are keyed to the Vita Classic Shade guide, but some brands distinguish shades according to value, hue or translucency. Moreover, large quantitative color differences have been detected among resin composites of the same Vita shade (27).

Regarding the variation of micro hardness values of each luting material tested according to the root third, all of them exhibited statistically higher micro hardness values in the cervical third. Previous works have determined that the tendency of degree of conversion to decrease within the root canal depends on the type of post (6,29,30,31), considering translucent posts the best option to enhance light transmission (4,6). No reference has been found regarding the ability of the posts tested to transmit light. However, Alves et al., (29) evaluated ten fiber posts of different manufacturers and translucencies, and for all posts a significant reduction in the quantity of light transmitted as the depth increased was revealed. According to these authors and Galhano et al., (30) the light transmission of the translucent posts in the deeper regions would be insufficient for clinical luminous activation of resin. Therefore, the three resin luting materials tested would still be dependent on light activation, with the proximity of the light curing unit playing a key role in their degree of conversion. The decrease in microhardness values from coronal to apical has been previously described for Calibra resin cement (31). It has been reported by Arrais et al., (25) that the fifth generation dual cure luting systems, as Calibra, contain a lower quantity of self-curing components that may not be capable of compensating for the decrease in light intensity.

On the other hand, Pedreira et al., (24) reported a homogeneous microhardness for RelyX Unicem along the root canal. However, methodological differences may have influenced as, in our study, the measurements were performed after 24 h and in their study after 7 days water storage, allowing a more intense influence of the root canal environment on RelyX Unicem curing.

The authors are aware this study focus in one type of fiber post, one light-curing procedure and only three resin luting materials. Therefore, the results obtained should not be extrapolated to other materials. Even so, according to our measurements, resin cement shade seems to be a less relevant factor on micro hardness. Finally, this study aims to determine micro hardness as an indirect measurement of curing degree and no value has been establish to ensure clinical success. Therefore, high micro hardness values are not necessarily related with a good clinical performance.
